# Biomechanical Evaluation of Seven Fixation Methods for Sagittal Split Ramus Osteotomy with Four Advancement Levels by Finite Element Analysis

**DOI:** 10.3389/fsurg.2022.891747

**Published:** 2022-05-04

**Authors:** Yu He, Henglei Zhang, Jia Qiao, Xi Fu, Shixing Xu, Qi Jin, Jianfeng Liu, Ying Chen, Bing Yu, Feng Niu

**Affiliations:** Department of Craniomaxillofacial Surgery, Plastic Surgery Hospital, Chinese Academy of Medical Sciences and Peking Union Medical College, Beijing, China

**Keywords:** sagittal split ramus osteotomy, rigid internal fixation, biomechanical characteristics, finite element analysis, plastic surgery

## Abstract

**Background:**

Mandibular sagittal split ramus osteotomy (SSRO) is a routine surgery to correct mandibular deformities, such as mandibular retrusion, protrusion, deficiency, and asymmetry. However, nonunion/malunion of the fragments and relapse caused by fixation failure after SSRO are major concerns. Rigid fixation to maintain postosteotomy segmental stabilization is critical for success. Additionally, understanding the biomechanical characteristics of different fixation methods in SSRO with large advancements is extremely important for clinical guidance. Therefore, the aim of the present study was to evaluate the biomechanical characteristics of different SSRO methods by finite element analysis.

**Methods:**

SSRO finite element models with 5-, 10-, 15-, and 20-mm advancements were developed. Seven fixation methods, namely, two types of bicortical screws, single miniplate, dual miniplates, grid plate, dual L-shaped plates, and hybrid fixation, were positioned into the SSRO models. Molar and incisal biomechanical loads were applied to all models to simulate bite forces. We then investigated the immediate postoperative stability from four aspects, namely, the stability of the distal osteotomy segment, osteotomy regional stability, stress distribution on the mandible, and implant stress performance.

**Results:**

The stability of the distal osteotomy segment and osteotomy region decreased when the advancement increased. All seven fixation methods displayed favorable biomechanical stability under minor advancement (5 mm). With large advancements, bicortical screws, dual miniplates, and grid plates provided better stability. The von Mises stress was concentrated around the screws close to the osteotomy region for the proximal segment for all fixation methods, and the von Mises stress on implants increased with larger advancements. With small advancements, five fixation methods endured tolerable maximum stresses of <880 MPa. A single miniplate and dual L-shaped plates generally suffered high stresses using larger advancements. The biomechanical characteristics were similar under molar and incisal loads.

**Conclusions:**

The current study investigated the biomechanical properties of seven fixation devices after SSRO under molar and incisal loads. Generally, bicortical screws, grid plates, and dual miniplates provided better biomechanical stability using finite element analysis.

## Background

The development of mandibular sagittal split ramus osteotomy (SSRO) is considered a milestone in craniomaxillofacial surgery (1). Sagittal osteotomy enables broad contact between bone segments for accurate deformity correction and apparent spatial change. After decades of continuous improvements, SSRO has become a routine surgery to correct mandibular deformities, such as mandibular retrusion, protrusion, deficiency, or asymmetry. SSRO also improves obstructive sleep apnea (OSA) through physical expansion of the pharyngeal tissues (2, 3). However, surgical complications, such as internal fixation failure, unfavorable splits, and temporomandibular joint dysfunction, result in unsuccessful outcomes (4, 5). Among the complications after SSRO, nonunion/malunion of the fragments and relapse caused by fixation failure are major concerns (6). The gap between the proximal and advanced distal segments makes the osteotomy site unstable. Therefore, rigid fixation to maintain postosteotomy segmental stabilization is critical for success.

Rigid internal fixation has been advocated to secure osteotomy segments, prevent relapse, and promote bone healing after SSRO (7). Several methods have been used to achieve rigid fixation, including bicortical screws, miniplates, and hybrid fixation (8–10). Bicortical screw fixation is considered the most cost-effective method, with ideal biomechanical stability (11). However, potential nerve injury, condylar displacement, and the need for additional extraoral incisions limit the clinical use of bicortical screws. A miniplate and monocortical screw fixation system is associated with a low risk of nerve injuries and allows operation through sole intraoral incisions. However, rigid fixation is difficult to obtain using only a single miniplate. To overcome the shortcomings, derivative methods are used to increase stabilization, such as dual miniplates, Y-shaped plates, dual L-shaped plates, grid plates, and hybrid techniques (6, 7, 9, 12–15). Hybrid fixation was advocated by Schwartz and Relle to combine the advantages of bicortical screws and miniplates with monocortical screws (15). Some biomechanical studies have proven that hybrid techniques provide ideal biomechanical stability (8, 16).

Previous biomechanical studies usually used a 5-mm advancement of the distal segment in SSRO to evaluate stability. However, in craniomaxillofacial practice, 10-mm or even 20-mm advancement is not uncommon for severe deformities (17), and a large advancement of >10 mm is necessary for the treatment of OSA to achieve airway improvement (2, 18). However, the unsatisfactory bone contact caused by large advancement leads to extreme instability at the osteotomy site. Rigid fixation is particularly important in large advancements to maintain stabilization and prevent relapse. Understanding the biomechanical characteristics of different fixation methods in SSRO with large advancements is of great importance for clinical guidance. Therefore, the aim of the present study was to evaluate the biomechanical characteristics of seven fixation methods in SSRO with 5–20-mm advancements under molar and incisal loads by finite element analysis.

## Methods

### Finite Element Models

**Figure 1:**
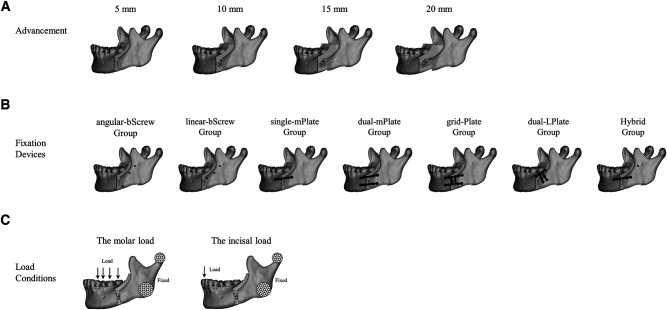
Mandibular sagittal split ramus osteotomy was performed on the intact mandible models. The forward advancement of the distal segment was set up to 5, 10, 15, and 20 mm (**A**). Seven types of fixation methods were positioned in the osteotomy models (**B**). Molar and incisal loads were applied to all models to simulate bite forces (**C**).

Finite element models were developed using computed tomography (Lightspeed VCT; GE, Fairfield, CT, USA) images from 10 healthy volunteers. SSRO was performed on the intact mandible models. The forward advancement of the distal segment was set to 5, 10, 15, and 20 mm. The implants for seven fixation methods were positioned in the SSRO models according to standard operative guidelines (**Figure 1**). The implants and fixation methods comprised bicortical screws with an angular arrangement (angular-bScrew), bicortical screws with a linear arrangement (linear-bScrew), single miniplate with monocortical screws (single-mPlate), dual miniplates with monocortical screws (dual-mPlate), grid plate (grid-Plate), dual L-shaped plates (dual-LPlate), and hybrid fixation using one bicortical screw and one miniplate (Hybrid). Using bicortical screws and dual L-shaped plates was not an option when advancement was 20 mm because of the limited fixation space. The length and diameter of the bicortical screws were 12 and 2 mm, respectively. The miniplates were fixed using 2.0 mm × 6.0 mm monocortical screws. The cortical bone and cancellous bone were assumed to have a Poisson’s ratio of 0.3 and elastic moduli of 14,000 and 1,500 MPa (6), respectively. The implants of the different fixation devices were set at a Poisson’s ratio of 0.34 and an elastic modulus of 114,000 MPa (6). The contact interface of the screw and plate was set as a rigid bond, and the screws were locked to the mandible. The screw threads were omitted to simplify the models, and linear elastic isotropic material properties were assigned to the SSRO models and internal fixation devices.

### Finite Element Analysis

Abaqus 6.13 (3DS, Waltham, MA, USA) was used to perform the finite element analysis. The SSRO models with the four levels of advancement were fixed using the seven fixation methods (*n* = 10). Two biomechanical loads were applied to all models to simulate bite forces (**Figure 1**). A 500-N molar load was applied to the posterior teeth perpendicular to the occlusal plane. The components of the molar force on the first premolar, second premolar, first molar, and second molar were 50, 100, 250, and 100 N, respectively. The incisal load was defined as a vertical 150-N load that acted on the incisal edge. The mandibular condyle and lateral ramus were fixed to represent the reaction force at the temporomandibular joint.

We investigated the immediate postoperative stability from four aspects, namely, the stability of the distal osteotomy segment, osteotomy regional stability, stress distribution on the mandible, and stress performance of the seven internal fixation devices. The stability of the distal osteotomy segment under molar and incisal loads was expressed by the construct stiffness. The osteotomy regional stability was determined by the displacement variation between the proximal and distal osteotomy segments. The distribution of von Mises stress on the mandible was displayed to understand the mechanical transmission characteristics, and the peak value and distribution of von Mises stress on the fixation devices were assessed to understand the force features.

### Statistical Analysis

Statistical analyses were performed using SPSS software (version 19.0; IBM Corp., Armonk, NY, USA). A separate one-way analysis of variance was performed to compare the average values of the different groups, and *p*-values <0.05 were considered statistically significant.

## Results

### Stability of the Distal Osteotomy Segment

**Figure 2:**
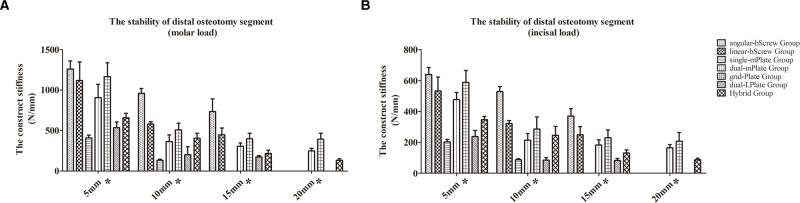
Construct stiffness outcomes of the distal osteotomy segment under molar (**A**) and incisal (**B**) load conditions. *p < 0.05.

The construct stiffness outcomes of the distal osteotomy segment under different load conditions are shown in **Figure 2**. Generally, the stability of the distal osteotomy segment decreased when the advancement increased. The bicortical screws and grid plates achieved better construct stiffnesses than that with other fixation methods with a 5 mm advancement. With 10 and 15 mm advancements, angular-bScrew fixation showed better results than linear-bScrew fixation. The finite element analysis process was not convergent when the osteotomized mandible was fixed with a single miniplate under 15 and 20 mm advancements. Under molar and incisal loads, the characteristics of the construct stiffness after different fixation methods were similar.

### Osteotomy Regional Stability

**Figure 3:**
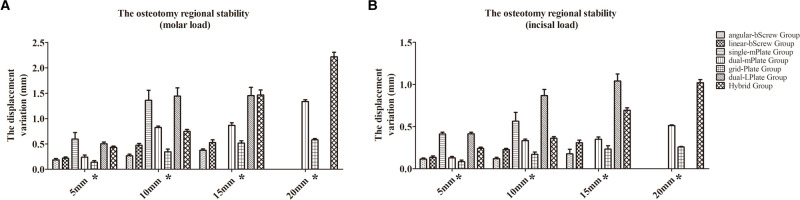
Results of the osteotomy regional stability after different fixation methods under molar (**A**) and incisal (**B**) load conditions. *p < 0.05.

The results of the osteotomy regional stability are shown in **Figure 3**. The osteotomy regional stability decreased as the advancement increased. With a 5 mm advancement, satisfactory results were obtained regarding the displacement variation between the proximal and distal osteotomy segments under molar loading (from 0.137 ± 0.033 mm to 0.595 ± 0.128 mm) and incisal loading (from 0.083 ± 0.018 mm to 0.414 ± 0.019 mm). When the advancement was >5 mm, the bicortical fixation methods and grid plates were associated with less displacement variation. The maximum value (2.220 ± 0.086 mm) of the displacement variation appeared in the Hybrid group with a 20 mm advancement under molar loading.

### Stress Distribution on the Mandible

**Figure 4:**
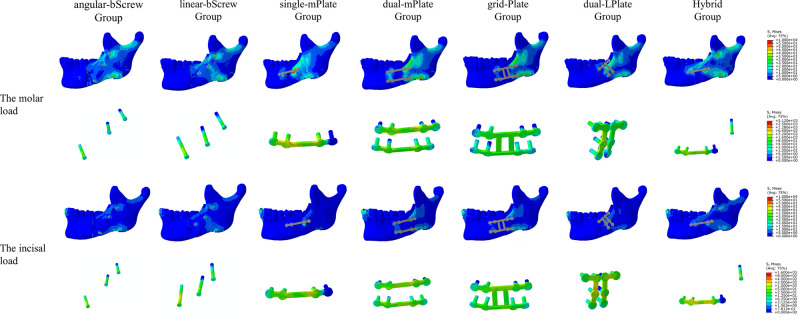
Distribution of von Mises stress on mandibles and the von Mises stresses results for seven fixation methods with a 5 mm advancement.

**Figure 5:**
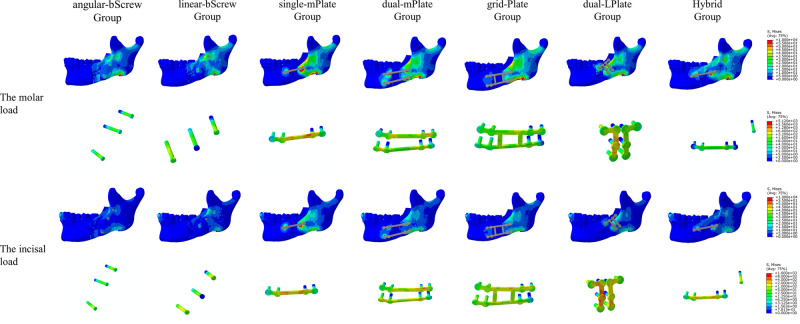
Distribution of von Mises stress on mandibles and the von Mises stresses results for seven fixation methods with a 10 mm advancement.

**Figure 6:**
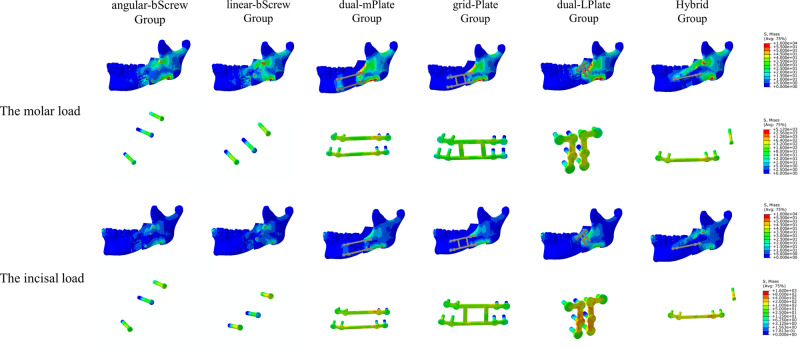
Distribution of von Mises stress on mandibles and the von Mises stresses results for seven fixation methods with a 15 mm advancement.

**Figure 7:**
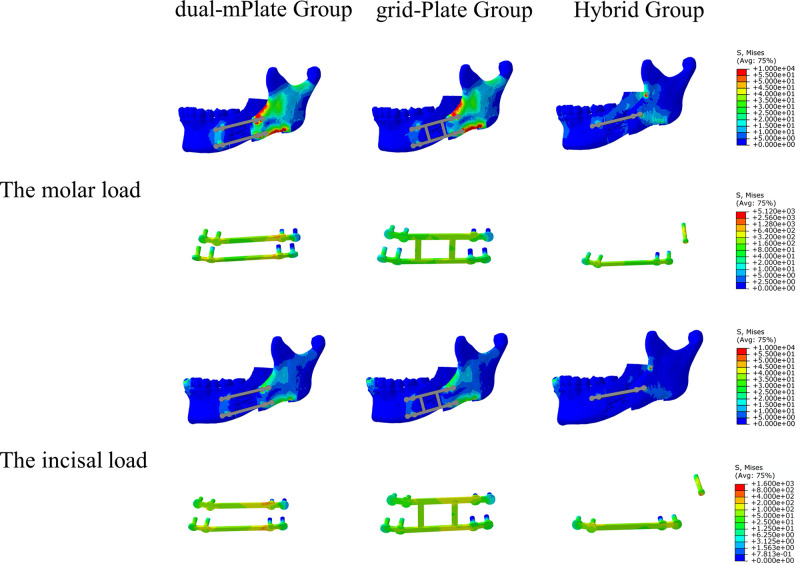
Distribution of von Mises stress on mandibles and the von Mises stresses results for seven fixation methods with a 20 mm advancement.

The distribution of von Mises stress on the mandible is shown in **Figures 4–7**. For the proximal segment, the von Mises stress was concentrated around the screws close to the osteotomy region for all groups. The von Mises stress dissipated to the upper and posterior areas of the segment. For the distal segment, the von Mises stress was concentrated at the screw-bone interface near the osteotomy site. With single-mPlate, dual-mPlate, and Hybrid fixation methods, the stresses concentrated around the implants were higher than the stresses with other fixation methods. The stress on the distal segment was higher with dual-mPlate fixation than that with grid-Plate fixation, with larger advancements. Bicortical screw fixation was associated with lower concentrated von Mises stress. With a 20 mm advancement, the von Mises stress was obviously less with Hybrid fixation than that with dual-mPlate and grid-plate fixation. The features of the stress distribution on the mandible were similar under molar and incisal loads.

### Stress Performance of the Fixation Devices

**Figure 8:**
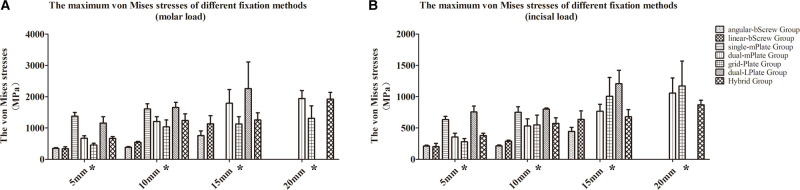
Maximum von Mises stresses of seven fixation methods under molar (**A**) and incisal (**B**) load conditions. *p < 0.05.

The von Mises stress results for the seven fixation methods are shown in **Figures 4–8**. The von Mises stress on implants increased with larger advancements. With a 5 mm advancement, the angular-bScrew (349.625 ± 23.907 MPa), linear-bScrew (341.845 ± 60.538 MPa), dual-mPlate (627.319 ± 81.498 MPa), grid-Plate (460.313 ± 53.135 MPa), and Hybrid implants (666.949 ± 58.228 MPa) endured tolerable maximum stresses of <880 MPa. Only the bicortical screws withstood less von Mises stress with 5, 10, and 15 mm advancements. With larger advancements, a single miniplate and dual L-shaped plates generally suffered high stresses. With fixation, high von Mises stress appeared at the plate–screw junction and near the osteotomy region. Compared with dual-mPlate and single-mPlate fixation, the von Mises stresses concentrated on the implants were dispersed with grid-Plate and Hybrid fixation.

## Discussion

Immediate postoperative stability after SSRO is closely related to the incidence of complications and healing time. Several fixation methods are used to enhance stability, such as bicortical screws, miniplates, dual L-shaped plates, grid plates, and hybrid fixation. In the current study, the biomechanical characteristics of seven fixation methods in SSRO with 5–20-mm advancements were evaluated by finite element analysis. The seven fixation methods displayed favorable biomechanical stability with a minor advancement of 5 mm. With large advancements, bicortical screws, dual miniplates, and grid plates provided better stability.

The biomechanical characteristics of different fixation methods in SSRO have been explored in previous studies (6–10, 12, 13, 17, 19–23); however, these studies focused mainly on small advancements (7, 9, 10, 13, 19, 22, 23). In clinical practice, large advancement in SSRO is not uncommon to correct severe mandibular deformity and improve the airway in OSA (17, 18). Therefore, it is necessary to understand the biomechanical stabilization of different fixation methods in SSRO under large advancements. Klein et al. used six different devices to fix osteotomy segments, with a 10-mm advancement (12). The results showed that dual miniplates provided the least amount of displacement. De Oliveira et al. created SSRO models with an 8-mm gap at the upper border and an 11-mm gap at the lower border of the distal segment to simulate mandibular advancement with counterclockwise rotation (17). Six fixation systems were used to fix the osteotomy segments. The authors found that two four-hole miniplates and one eight-hole grid miniplate provided stronger resistance forces. In our study, we evaluated seven fixation devices to fix the osteotomy segments with large advancements. The results showed that bicortical screws, dual miniplates, and grid plates provided better biomechanical stability with different advancements. However, the finite element analysis process was not convergent when the osteotomized mandible was fixed with a single miniplate under 15 and 20 mm advancements. The unexpected results suggested that with these advancements, the osteotomy segments were extremely unstable. With a 20 mm advancement, we could not use bicortical screws and dual L-shaped plates because of limited fixation space. These results provide tips for the clinical application of fixation in SSRO with large advancement.

Bicortical screws are considered the optimal fixation method (24). However, the configuration of bicortical screws is diverse. One study found no statistical difference in biomechanical strength between three bicortical screws placed at 90° and 60° angles (25). Another study reported better load resistance at a 90° angle than that at a 60° angle (26). Our study revealed that bicortical screws showed similar biomechanical properties with small advancements. With >10 mm advancements, the biomechanical performance of bicortical screws with an angular arrangement was better than with a linear arrangement by finite element analysis. Additionally, bicortical screws were the ideal fixation devices after SSRO, with higher construct stability and lower local displacement than with other fixation methods. The single miniplate showed the worst results regarding the least amount of displacement in the osteotomy region. To prevent relapse, dual miniplates, grid plates, Y-shaped plates, dual L-shaped plates, and hybrid fixation could be considered improved fixation technology over single miniplate (6, 7, 9, 12–14). Several studies investigated the effect of different types of miniplates on fixation in SSRO. These studies found that dual miniplates provided better biomechanical stability than a single miniplate and dual Y-shaped plates (12, 17). However, the fixation effect between dual miniplates and grid plates after SSRO is disputed. Sener et al. found that grid plates provided better load resistance than dual miniplates (27). Oguz et al. and De Oliveira et al. suggested that the two fixation methods provided the same degree of osteotomy segmental stability (10, 17). Our study showed better results regarding distal osteotomy segment stability and regional displacement with grid-Plate than with dual-mPlate fixation. However, the von Mises stress on the grid plates was less than that on the dual miniplates. We believe that grid plates resist loads and disperse stress.

Novel fixation devices have been developed and tested with the goal of providing stronger segmental stability after SSRO. Sonego et al. introduced a new method of fixing osteotomy segments with dual L-shaped plates (9). Using biomechanical tests, the authors found that dual L-shaped plates and bicortical screws provided similar stability after SSRO, and the stability was greater than that with straight titanium plates. The authors believed that dual L-shaped plates contributed to a favorable distribution of both convergent and divergent stresses by being fixed on the external surface of the proximal segment vertically and on the distal segment horizontally. In our study, fixation with dual L-shaped plates provided unsatisfactory intact construct stiffness and the plates endured high von Mises stress. Considering the limitations of finite element analysis, a more detailed biomechanical evaluation of this novel fixation method is necessary before clinical application. Schwartz and Relle introduced hybrid fixation, combining the advantages of bicortical screws and miniplate with monocortical screws (15). Several studies reported that this hybrid technique was a promising clinical device, with suitable strength and preferable operability (8, 10, 23). From a biomechanical view, hybrid fixation was associated with less stability than bicortical screws. In our study, similar results regarding hybrid fixation were observed, with this method providing better stability than that with single miniplates and dual L-shaped plates, although the method was not as strong as other fixation methods.

In biomechanical research, the molars and incisors forces are the main loading methods to investigate the characteristics of SSRO fixation. The influence of different loading methods on the research results has not been deeply studied. In the current study, the molar and incisal loads were applied to all models to simulate bite forces. A significant difference was not observed between the two load methods, including stability of the distal osteotomy segment, osteotomy regional stability, stress distribution on the mandible, and stress performance of the fixation methods. We inferred that two loading methods were suitable for the present study and produced similar effects. The difference between the molar and incisal loads for finite element analysis should be further investigated.

There are limitations in the present study. First, the finite element analysis was mainly based on the mandible, similar to other finite element studies. The current research strategy neglected the influence of maxilla and soft tissue on the biomechanical results. Second, the biomechanical loading methods on the mandible were inconsistent. After the pretest and literature review, we chose molar and incisal loads to simulate the physiological state of the mandible as much as possible. The effect of postoperative maxillomandibular fixation on the stability of different fixation devices was not considered. Third, the present study analyzed only the results from a biomechanical perspective without considering clinical usefulness. For example, we believe that bicortical screws provide the best biomechanical stability compared with other fixation methods. However, the potential clinical disadvantages of bicortical screws were not considered, such as compression of the inferior alveolar nerve, condylar torque, and the additional extraoral approach. Overall, the results only provided biomechanical clues for clinical application. The specialized treatment plans should be made by craniomaxillofacial surgeons according to the specific situation in clinical practice, such as morphological characteristics of malformation, postoperative maxillomandibular fixation strategy, and surgeons’ experience. Therefore, the conclusions of the current study should be interpreted carefully before the clinical application of the fixation methods.

## Conclusions

In conclusion, the current study investigated the biomechanical properties of seven fixation devices in SSRO under molar and incisal loads. Generally, bicortical screws, grid plates, and dual miniplates provided better biomechanical stability in finite element analysis.

## Data Availability

The raw data supporting the conclusions of this article will be made available by the authors without undue reservation.
